# Granulomatosis With Polyangiitis Presenting as Chest Pain: A Case Report

**DOI:** 10.7759/cureus.38204

**Published:** 2023-04-27

**Authors:** Kindalem Fentie, Swathi Singanamala, Omar Hozayen, Lareb Altaf

**Affiliations:** 1 Medicine, Trinity Medical Sciences University, Warner Robins, USA; 2 Nephrology, Piedmont HealthCare, Macon, USA

**Keywords:** kidney biopsy, wegener’s granulomatosis, pauci-immune crescentic glomerulonephritis, non-st-segment elevation myocardial infarction (nstemi), unusual causes of chest pain, granulomatosis with polyangiitis (gpa)

## Abstract

Granulomatosis with polyangiitis (GPA) is a relatively rare systemic autoimmune disorder of small and medium size blood vessels affecting multiple organs with a wide range of clinical presentations. We present a 57-year-old Caucasian male who presented to the ER with midsternal chest pain. He was hospitalized for non-ST elevated myocardial infarction (NSTEMI) and later diagnosed with pauci-immune necrotizing crescentic glomerulonephritis confirmed with renal biopsy.

## Introduction

Granulomatosis with polyangiitis (GPA) is a vasculitis characterized by necrotizing granulomas of the nasopharynx, lungs, and kidneys. It classically presents in middle-aged males as a triad of sinusitis or nosebleeds, hemoptysis with bilateral nodular lung infiltrates, and hematuria due to rapidly progressive (crescentic) glomerulonephritis. Additionally, laboratory findings typically present with a positive cytoplasmic-antineutrophil cytoplasmic autoantibody (c-ANCA) [1,2]. 

GPA, formerly known as Wegener’s granulomatosis, is one of the three vasculitides associated with ANCA. The other two with this association are microscopic polyangiitis and eosinophilic granulomatosis with polyangiitis, formerly known as Churg-Strauss syndrome. GPA affects approximately three per 100,000 people in the United States, with 2300 diagnoses annually. The challenge in identifying GPA in patients stems from not only the relative rarity of the disease but also its varied clinical course that can affect nearly any organ system [3].

Due to the unobvious and diverse manifestations of GPA, there is currently no standardized clinical diagnostic framework for the disease. However, the c-ANCA test can establish GPA as the cause of a patient’s autoimmune presentation [3]. c-ANCA is an autoantibody that shows diffuse, granular staining within the neutrophilic cytoplasm under microscopy. While a positive test having 90% specificity would confidently indicate GPA, it should be noted that a negative test does not rule out the disease as there is only 75% sensitivity with the c-ANCA test [4].

Pauci-immune glomerulonephritis is an inflammatory kidney disease characterized by necrotizing glomerulonephritis with few or no immune complex deposition by immunofluorescence (IF) or electron microscope (EM). It is the most common type of rapidly progressive glomerulonephritis and is frequently associated with an ANCA [1].

## Case presentation

A 57-year-old Caucasian male with a past medical history of hypertension, hyperlipidemia, gastroesophageal reflux disease (GERD), emphysema, degenerative joint disease, and 30 pack-year smoking presented to the emergency department with 6/10 midsternal chest pain radiating to his left arm, which lasted for 10-15 minutes. The chest pain started at rest while in bed and was relieved by the placement of a Nitro patch. Troponin on admission was 0.268, which trended up to 5.006. He underwent left heart catheterization for non-ST elevated myocardial infarction (NSTEMI) after receiving intravenous fluids and N-acetylcysteine with percutaneous coronary intervention (PCI) x2. 

During his annual physical two weeks prior, he was diagnosed with acute renal failure by his primary care provider (PCP) with an elevated creatinine of 2.0 mg/dl. He was prompted to stop taking meloxicam and aspirin. 

Upon examination, he had an unremarkable physical exam and was hemodynamically stable (Table 1). Laboratory workup was notable for mild leukocytosis, low hemoglobin, abnormal renal function test, and positive ANCA (Table 2).

**Table 1:** Initial vital sign findings

Vital Sign	Patient’s finding
Temperature	98.6 °F
Heart rate	96 beats per minute
Blood pressure	131/86 mmHg
Respiratory rate	22 breaths per minute
Oxygen saturation	95% on room air

**Table 2:** Labratory test results

Laboratory tests	Lab Result	Normal Range
White blood count	11.53 x10^9^/µL	4.0-10 x10^9^/µL
Hemoglobin	12.3 g/dL	14-17 g/dL
Sodium	135 mmol/L	136-145 mmol/L
Potassium	4.3 mmol/L	3.5-5.0 mmol/L
Serum creatinine	3.81 mg/dL	0.7-1.3 mg/dL
Blood urea nitrogen	68 mg/dL	8-20 mg/dL
Random blood glucose	136 mg/dL	70-140 mg/dL
Complement component 3 factor	201 mg/dL	80 – 178 mg/dL
Complement component 4 factor	37 mg/dL	12 – 42 mg/dL
Anti-neutrophil cytoplasmic antibody	Positive	Negative

He underwent a renal biopsy with both light microscopy (LM) and IF. The LM demonstrated five glomeruli that depicted one glomerulus with global sclerosis and two glomeruli with fibro-cellular crescents. The IF demonstrated no linear anti-glomerular basement membrane (anti-GBM) staining for IgG. There was 1+ segmental granular mesangial and capillary loop staining near the sclerotic segment for IgG, IgA, and IgM via IF but without immune complex deposition via EM. Together with ANCA positivity, these findings were characteristics of pauci-immune necrotizing crescentic glomerulonephritis.

He was treated with prednisone for two months while waiting for a renal biopsy. Once a renal biopsy confirmed the diagnosis of pauci-immune necrotizing crescentic glomerulonephritis, he was admitted and treated with IV cyclophosphamide. This led to a significant improvement in kidney function with creatinine levels trending down to 1.7 mg/dL. The patient was discharged home with maintenance therapy of azathioprine and a close follow-up with a nephrologist. 

## Discussion

Pauci-immune glomerulonephritis is a histopathological diagnosis of glomerulonephritis associated with a group of vasculitis, including GPA, microscopic polyangiitis, and eosinophilic granulomatosis with polyangiitis, which are characterized by necrotizing crescentic GN with few or no immune complex deposition via IF or EM on biopsy.

GPA is a systemic autoimmune vasculitis that affects both medium and small-sized vessels. First and foremost, these patients typically present with symptoms of local inflammation, initially with upper respiratory tract involvement in 92% of cases, often noting the first clinical manifestation includes chronic sinusitis, epistaxis, or otitis media. Second, they have lower respiratory tract symptoms with involvement of the lungs in 85% of cases. Lastly, there is renal involvement in 77% of cases. Unlike the typical presentation, our patient presented with chest pain later in the clinical course of the disease [5].

GPA can present with late complications of vital organ involvement. In a cohort study of 517 patients with GPA followed for nine years, cardiac involvement was found in 3.3 %, of which pericarditis was the most common, followed by cardiomyopathy, coronary artery disease (CAD), and valvular disease, which could present as chest pain or other cardiac symptoms. The cardiac presentation was noted in our patient, who presented with chest pain and was later diagnosed as NSTEMI. It is challenging to identify the cause of CAD in GPA patients with multiple risk factors versus those who solely have coronary artery vasculitis secondary to GPA. In our patient, he had other risk factors for CAD, with smoking being the most significant risk factor, which makes it challenging to identify the cause of his NSTEMI, whether it was purely secondary to GPA versus secondary to his other risk factor [2].

GPA often presents with renal vasculitis. In fact, 80% of GPA patients develop renal involvement within two years of the disease onset. The most common renal presentation is rapidly progressive glomerulonephritis (RPGN), which can lead to renal failure [6]. As with our patient, the RPGN is pauci-immune, indicating it is not associated with immune-complex deposition under IF. Instead, under a microscope, a segmental sclerotic necrotizing pattern can typically be visualized; this is consistent with our patient’s kidney biopsy. These findings culminated in causing the patient to present with signs of renal failure, including elevated creatinine and a declining glomerular filtration rate (GFR) [7].

## Conclusions

Chest pain is an unusual presentation for GPA, as depicted in our patient’s presentation. Hence it is imperative to have a high index of suspicion to diagnose GPA due to the wide range of clinical presentations. Therefore, it is beneficial to include GPA in the differential diagnosis for patients presenting with chest pain and include immunological tests like ANCA earlier in the workup for early diagnosis and treatment of GPA, which could result in better treatment outcomes.
